# Quantitative analysis of the dexamethasone side effect on human-derived young and aged skeletal muscle by myotube and nuclei segmentation using deep learning

**DOI:** 10.1093/bioinformatics/btae658

**Published:** 2025-01-03

**Authors:** Seonghwan Park, Min Young Kim, Jaewon Jeong, Sohae Yang, Minseok S Kim, Inkyu Moon

**Affiliations:** Department of Robotics and Mechatronics Engineering, DGIST, Daegu, 42988, South Korea; Department of New Biology, DGIST, Daegu, 42988, South Korea; Department of Robotics and Mechatronics Engineering, DGIST, Daegu, 42988, South Korea; Department of New Biology, DGIST, Daegu, 42988, South Korea; Department of New Biology, DGIST, Daegu, 42988, South Korea; CTCELLS Inc., Seoul, 06307, South Korea; Department of Robotics and Mechatronics Engineering, DGIST, Daegu, 42988, South Korea

## Abstract

**Motivation:**

Skeletal muscle cells (skMCs) combine together to create long, multi-nucleated structures called myotubes. By studying the size, length, and number of nuclei in these myotubes, we can gain a deeper understanding of skeletal muscle development. However, human experimenters may often derive unreliable results owing to the unusual shape of the myotube, which causes significant measurement variability.

**Results:**

We propose a new method for quantitative analysis of the dexamethasone side effect on human-derived young and aged skeletal muscle by simultaneous myotube and nuclei segmentation using deep learning combined with post-processing techniques. The deep learning model outputs myotube semantic segmentation, nuclei semantic segmentation, and nuclei center, and post-processing applies a watershed algorithm to accurately distinguish overlapped nuclei and identify myotube branches through skeletonization. To evaluate the performance of the model, the myotube diameter and the number of nuclei were calculated from the generated segmented images and compared with the results calculated by human experimenters. In particular, the proposed model produced outstanding outcomes when comparing human-derived primary young and aged skMCs treated with dexamethasone. The proposed standardized and consistent automated image segmentation system for myotubes is expected to help streamline the drug-development process for skeletal muscle diseases.

**Availability and implementation:**

The code and the data are available at https://github.com/tdn02007/QA-skMCs-Seg

## 1 Introduction

Skeletal muscle plays a crucial role in the body by facilitating movement and supporting various metabolic functions (Nair 2005, Jeong *et al.* 2022). These skeletal muscles originate from myoblasts, which are specialized precursor cells. During the process of myogenesis, myoblasts differentiate and fuse to form multinucleated structures called myotubes. This fusion is essential for the growth and repair of muscle tissue. Myotubes eventually mature into fully developed muscle fibers that are capable of contraction and generating force (Frontera and Ochala 2015). The formation and proper function of skeletal muscle cells (skMCs) are vital not only for locomotion but also for maintaining posture, stabilizing joints, and generating heat during physical activity. Thus, skMCs are integral to both everyday activities and overall metabolic health (Kim *et al.* 2023).

Dexamethasone, a synthetic glucocorticoid, is commonly prescribed to treat a variety of inflammatory and allergic diseases, such as rheumatoid arthritis, asthma, and lupus (Qin *et al.* 2013, Otsuka *et al.* 2019). However, dexamethasone induces muscle atrophy by promoting catabolic pathways that lead to protein degradation and by inhibiting anabolic pathways responsible for protein synthesis (Jesinkey *et al.* 2014). Specifically, dexamethasone activates the ubiquitin−proteasome system and autophagy−lysosome pathways, which degrade myofibrillar proteins, while concurrently suppressing the mTOR signaling pathway, which is crucial for muscle protein synthesis. These adverse effects of dexamethasone are particularly pronounced in the elderly, where muscle wasting, also known as sarcopenia, is already prevalent due to age-related declines in anabolic hormone levels and physical activity (Seene and Kaasik 2012). Consequently, prescribing dexamethasone to elderly patients can exacerbate sarcopenia, leading to severe complications such as reduced mobility, increased frailty, and higher susceptibility to falls and fractures. Moreover, muscular atrophy in aged individuals diminishes metabolic efficiency, impairs immune function, and increases mortality rates (Brown *et al.* 2016, Toptas *et al.* 2018, Sobestiansky *et al.* 2019). Therefore, it is imperative to prevent dexamethasone-induced atrophy in elderly populations.

Various conditions, such as sarcopenia, muscular dystrophy, and cachexia, result in muscular diseases that significantly impair quality of life and lack effective pharmacological treatments (Walston 2012, Kim *et al.* 2020). Sarcopenia is characterized by the progressive loss of skeletal muscle mass and function with age, while muscular dystrophies are a group of genetic disorders that cause muscle weakness and degeneration. Cachexia, often associated with chronic illnesses such as cancer, heart failure, and chronic obstructive pulmonary disease, leads to severe muscle wasting and weight loss. Despite ongoing research efforts, effective treatments for these conditions remain elusive (Morley 2018). Consequently, developing novel therapeutic strategies to combat muscle diseases is a primary focus for researchers and pharmaceutical companies (Kwak and Kwon 2019).

**Figure 1.**
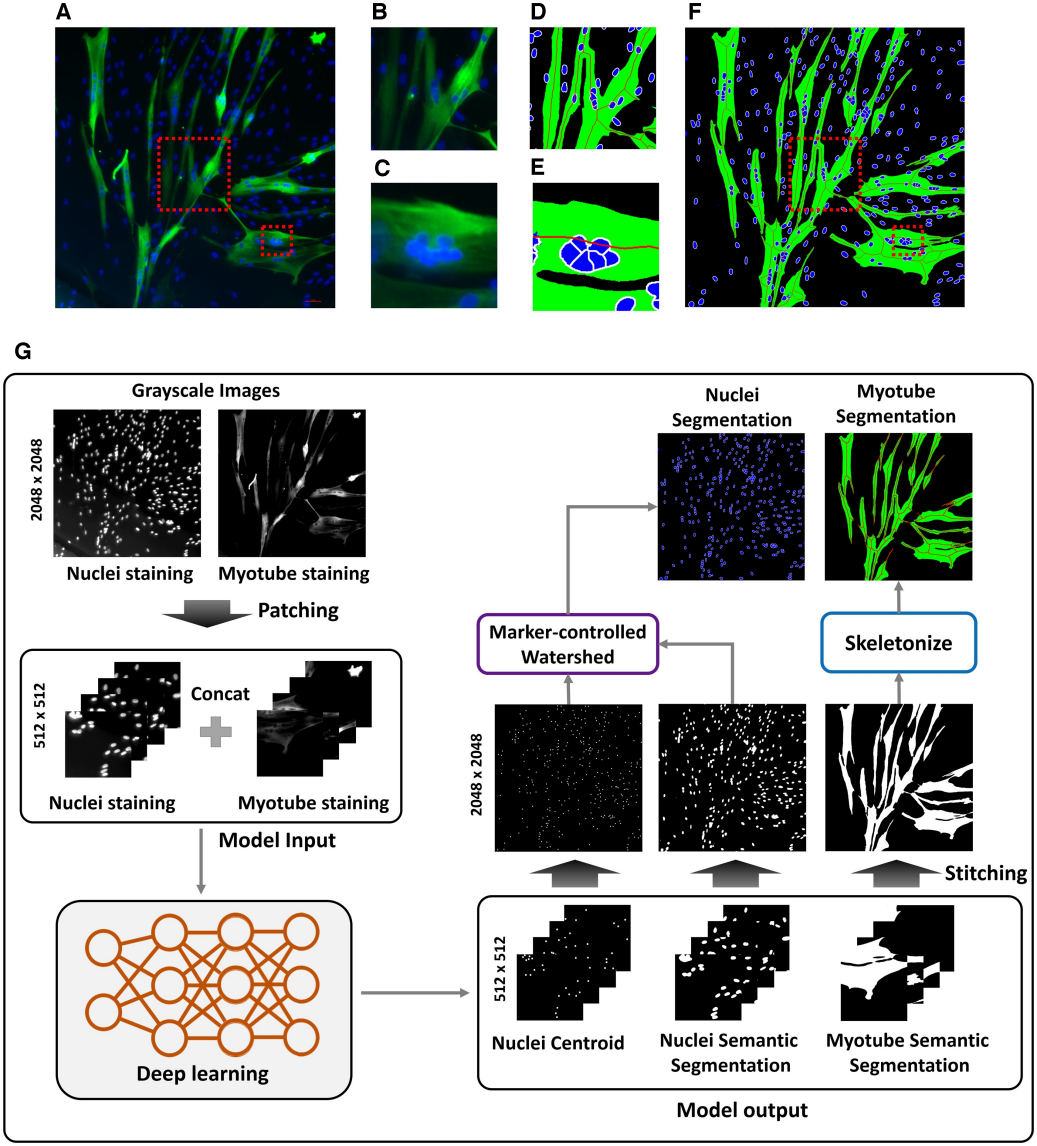
Process of performing segmentation from myotube (Myh1) and nuclei (DAPI) fluorescence images. (A) is the original fluorescence image. (B) and (C) are enlarged images of the box from image (A). (D) and (E) are images in which the box is enlarged from the (F) image generated by performing the (G) process. (G) is the process of generating the segmentation image (F) to calculate quantitative parameters of myotube through deep learning and post-processing from the original fluorescence image.

In the early stages of drug development, researchers typically screen compounds by analyzing their effects on cellular morphology or employing quantitative measures through image analysis (Chandrasekaran *et al.* 2021). In muscle research, myoblasts differentiate and fuse to form myotubes, which lack a fixed shape, making them difficult to analyze using conventional automated software (Carpenter *et al.* 2006, Poletti *et al.* 2012, Noe *et al.* 2022). As a result, measurements of myotube diameter, length, and the number of nuclei per myotube can vary significantly when assessed manually, reducing data reliability and reproducibility (Fig. 1A−C). Additionally, manual analysis by researchers is time-consuming and labor-intensive, rendering it impractical for high-throughput drug screening. Therefore, for image-based automated myotube analysis, it is essential to develop technologies that can identify the exact boundaries of the myotubes as well as clearly distinguish between overlapping myotubes and nuclei (Fig. 1D−F).

Deep learning techniques are used in various fields for image processing. The convolutional neural network (CNN) is a deep neural network that extracts important features from input data by applying different kernels (Krizhevsky *et al.* 2017). CNNs have been applied to many biomedical image segmentation problems (Sadanandan *et al.* 2017, Al-Kofahi *et al.* 2018, Ghaznavi *et al.* 2022, Kim *et al.* 2022, Wu *et al.* 2023, Xiao *et al.* 2023). Ronneberger *et al.* proposed a U-net model for medical image segmentation based on an encoder-decoder architecture (Ronneberger *et al.* 2015). Deep learning has also been used for the analysis of fluorescence images. A study on the segmentation of fluorescence images of nuclei was performed using the U-net model (Long 2020). A deep-learning-based nuclei segmentation approach has also been proposed, where the model was designed using image-style transfer to augment the training data (Hollandi *et al.* 2020). Li *et al.* proposed a deep convolutional neural network followed by post-processing for detecting and measuring the cross-sections of muscle fibers (Li *et al.* 2019). Gu *et al.* proposed the MyoV, an advanced tool using deep learning to accurately segment and quantify muscle fibers (Gu *et al.* 2024).

However, previous studies do not simultaneously segment the nuclei and myotube. When the model for nuclei segmentation and the model for myotube segmentation are trained separately, the training time increases, and two models must be run to obtain the results, so it is not suitable for real-time application. Additionally, segmenting nuclei and myotubes using only deep learning has limitations. The reason for the segmentation of the myotube is to measure the exact diameter of the myotube. However, because myotubes overlap as they grow, it is difficult to measure the exact diameter using only images of myotubes segmented by deep learning. When performing nuclei segmentation, it is very important to segment overlapped nuclei. Deep learning models are indeed showing great effectiveness in segmentation, but CNN used for image segmentation utilizes pooling layers to reduce the spatial dimensions of the image as it passes through the network. While pooling helps reduce computational requirements and achieve spatial invariance, it also reduces the resolution of the feature maps. This loss of resolution can lead to less precise delineation of nuclei boundaries in the segmentation output.

We propose a new method to simultaneously segment the myotubes and nuclei in a fluorescence image using deep learning and post-processing for quantitative analysis of the dexamethasone side effect on human-derived young and aged skeletal muscle (Fig. 1G). We designed our deep learning model to produce three outputs: myotube semantic segmentation, nuclei semantic segmentation, and nuclei centroid. The three generated images are converted into images for calculating quantitative parameters of myotube through post-processing. Counting the number of nuclei is achieved by applying a marker-controlled watershed algorithm (Beucher and Meyer 1992) to the nuclei centroid image and nuclei semantic segmentation, which are the results of a deep learning model. Applying the deep learning results to the marker-controlled watershed algorithm shows results that can clearly distinguish overlapped nuclei. To calculate the myotube diameter, the skeletonization process (Lee *et al.* 1994) is performed on the myotube semantic segmentation, which is the result of a deep learning model. The skeleton can identify the branches of the myotube by reducing the binary object to a one-pixel-wide representation. Based on this, accurate myotube diameter calculation is possible. To assess the model performance, in addition to calculating evaluation metrics, the myotube diameter and the number of nuclei were calculated from the generated segmented images and compared with results calculated by human experimenters. In particular, we treated human-derived primary young and aged skMCs with dexamethasone and analyzed the effects using the deep-learning model. We expect that the development of such an automated image analysis system that can measure each myotube's diameter, length, and number of nuclei in a standardized and consistent manner could help streamline the drug development process.

## 2 Materials and methods

### 2.1 Data generation

We acquired 80 fluorescence images (2048 × 2048 pixels); 40 were used for training, and the remaining 40 for testing. Samples were made using the same human-derived cells, with multiple samples tested. Each experimental condition was performed in triplicate, and 3–4 images were taken per well. Primary skMCs (male donors aged 17 and 68 years) were purchased from Cook Myosite (SK-1111, LOT-01052-17M, 01013-68M). Twenty images were acquired for each of the primary young and aged human skMCs and the corresponding dexamethasone-treated groups, of which 10 were used for training and 10 for testing. Details on cell culture are described in the Supplementary Information (SI Methods S1 and Fig. S1).

As shown in Fig. 1G, the fluorescence images of the myotubes and nuclei were normalized to grayscale and concatenated into two channels. These images were then cropped into patches of size 512 × 512 pixels to facilitate model training. To increase the data diversity, data augmentation was performed using the rotate function (90°, 180°, and 270°), resulting in a total of 2560 patches. The design model consisted of three types of labels, namely myotube semantic segmentation, nuclei semantic segmentation, and nuclei centroid. The myotube semantic segmentation and nuclei semantic segmentation were first subjected to Otsu thresholding followed by manual correction of the misalignments. The nuclei are then centered manually by dotting with a white pointer at their centroids according to expert judgment. All labeled images are composed of 0 s and 1 s denoting black and white pixels, respectively.

### 2.2 Deep learning model architecture

**Figure 2.**
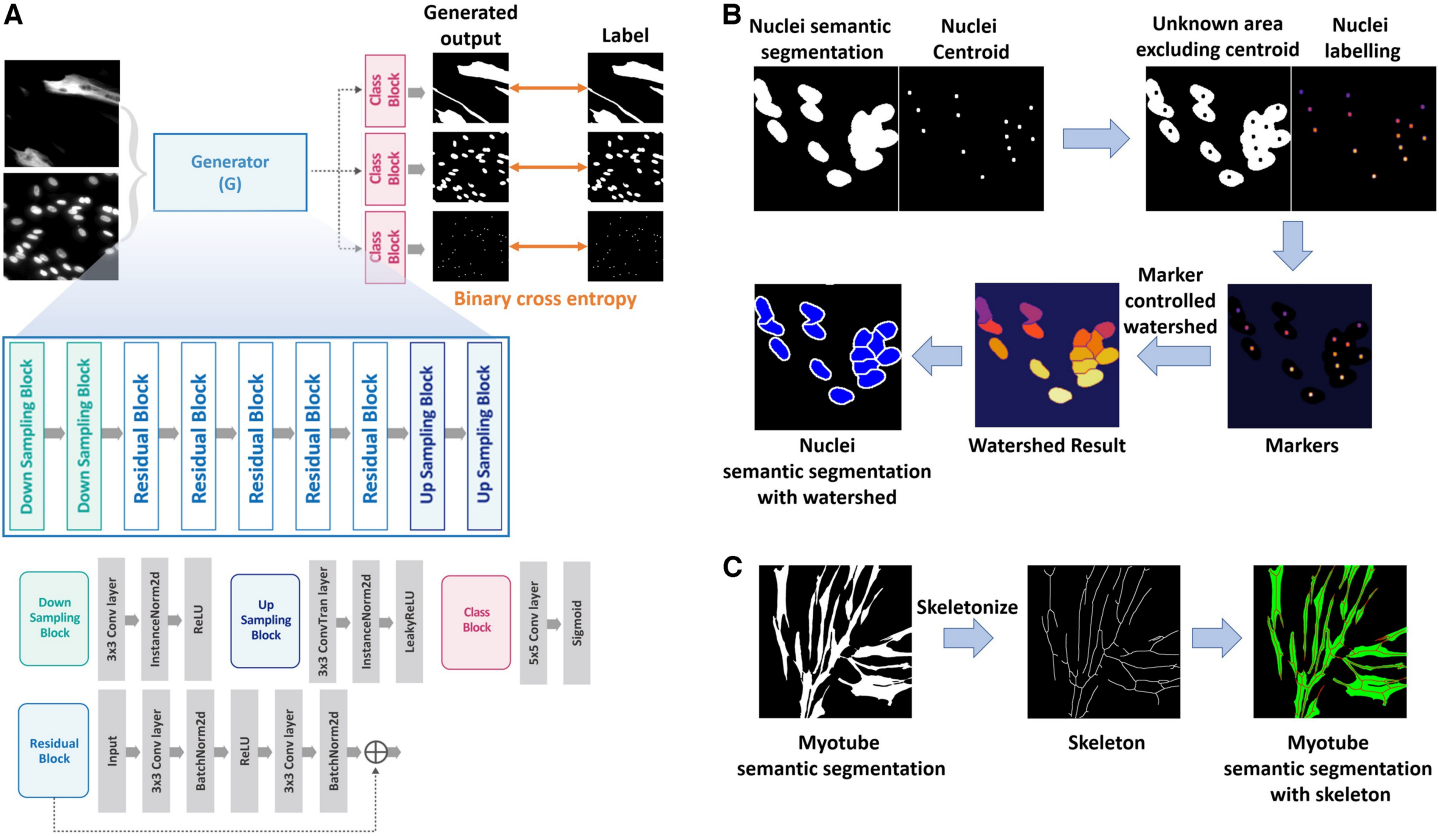
(A) is the architecture of the deep-learning model for myotube segmentation. (B) is the process of segmenting the nuclei by applying a marker-controlled watershed algorithm to nuclei semantic segmentation images and nuclei centroid images, which are the results of a deep learning model. (C) is the process of predicting the branch of each myotube by performing skeletonization from myotube semantic segmentation.

We propose a deep learning model that can simultaneously segment myotubes and nuclei based on the residual structure (Fig. 2A and Table 1). The model includes a generator consisting of downsampling for feature extraction through image size reduction, residual blocks for image analysis, and upsampling to reconstruct the original image size, and a class layer to generate three types of output. The downsampling operation extracts various features by doubling the channel dimension while reducing the image size to half by setting the stride value to two. We performed two rounds of downsampling in the model. The residual block analyzes the extracted features and generates an image corresponding to the result; the residual block uses skip connections for effective training (He *et al.* 2016). Residual blocks have the ability to alleviate the vanishing gradient problem and allow the network to learn deeper representations without losing information. By incorporating ID mapping, it allows the model to retain important low-level features, which helps in high-precision tasks such as image segmentation. Convolution, batch normalization, ReLU activation, convolution, and batch normalization are performed sequentially on the input to the residual block, and the sum of this processed output and input to the residual block is used as the final output. At this time, the summation operation is a skip connection. When a skip connection is used, the information generated by the block is matched with the input information to reduce losses. We used five residual blocks in the proposed model. Unlike downsampling, upsampling halves the channel dimension while doubling the image size. Similar to downsampling, upsampling was performed twice.

**Table 1.** Architecture of the deep-learning model for myotube segmentation.

Block	Layer	Output dimensions
Down Sampling Block 1	3 × 3 Conv (stride 2) Instance Norm ReLU	64 × 256 × 256
Down Sampling Block 2	3 × 3 Conv (stride 2) Instance Norm ReLU	128 × 128 × 128
Residual Blocks (x5)	3 × 3 Conv (stride 1) Batch Norm ReLU 3 × 3 Conv (stride 1) Batch Norm	256 × 128 × 128
Up Sampling Block 1	3 × 3 ConvTrans (stride 2) Instance Norm LeakyReLU	128 × 256 × 256
Up Sampling Block 2	3 × 3 ConvTrans (stride 2) Instance Norm LeakyReLU	64 × 512 × 512
Class Block 1	5 × 5 Conv (stride 1) Sigmoid	1 × 512 × 512
Class Block 2	5 × 5 Conv (stride 1) Sigmoid	1 × 512 × 512
Class Block 3	5 × 5 Conv (stride 1) Sigmoid	1 × 512 × 512

We added three separate blocks at the end of the generator structure as the class block. The result of upsampling was used as the input to each of the three blocks, resulting in three outputs, namely myotube segmentation, nuclei binary, and nuclei centroid. Using class-specific blocks further refines the output of each class, making the segmentation results more accurate and detailed, which helps the model produce more accurate boundaries and reduce class ambiguity in the segmented output. Each block comprises a convolutional layer and a sigmoid function.

### 2.3 Deep learning model objective

One of the goals of the proposed model was to find the weight values that minimized the loss functions between the three types of outputs and their corresponding ground truths. Each output is in the form of a binary image (0 s and 1 s). Therefore, the binary cross entropy was calculated between the ground truth and each generated output, and the model was trained in a direction in which the sum of the values was minimized.

Let x be a preprocessed fluorescence image used as the input image. When the generator including the downsampling block, residual block, and upsampling block is named G and the class block corresponding to each class k is named$C^{k}$, each output ${\hat{y}}^{k}$ can be defined as follows:

(1) $${\hat{y}}^{k}=C^{k}(G(x))$$

We applied binary cross entropy to each output ${\hat{y}}^{k}$. The calculation of binary cross entropy is as follows:

(2) $$L_{k}=-\frac{1}{N}\sum_{i=0}^{N}[y_{i}^{k}\cdot \;\text{log}({\hat{y}}_{i}^{k})+(1-y_{i}^{k})\cdot \;\text{log}(1-{\hat{y}}_{i}^{k})]$$

where $y^{k}$ is the ground truth; ${\hat{y}}^{k}$ is the generated output image corresponding to each class k; N is the number of pixels, and the average of the calculated differences for all pixels is designated as the loss. Therefore, the overall formula corresponding to the three outputs is as follows:

(3) $$L_{\mathrm{total}}=\lambda_{\mathrm{cls}1}L_{\mathrm{cls}1}+\lambda_{\mathrm{cls}2}L_{\mathrm{cls}2}+\lambda_{\mathrm{cls}3}L_{\mathrm{cls}3}$$

where λ is the weight of the loss corresponding to each output type, *cls*1 refers to myotube segmentation, *cls*2 refers to nuclei binary, and *cls*3 refers to the nuclei centroid. We used the Adam solver for optimization with adaptive momentum as well as *β*^1^ = 0.5 and *β*^2^ = 0.999 as the parameters. The learning rate was set to 0.001, and six graphics cards (NVIDIA RTX Quadro 6000) were used for the training. The training process required ∼12 h for 150 epochs.

### 2.4 Post-processing

In order to count the number of nuclei within a myotube, it is very important to distinguish between overlapped nuclei. However, it is difficult to distinguish the boundaries of the nuclei using only nuclei semantic segmentation generated through deep learning. We accurately distinguish overlapped nuclei by applying a marker-controlled watershed algorithm to the deep learning results, nuclei semantic segmentation and nuclei centroid (Fig. 2B).

First, the area that actually needs to be segmented, excluding the nuclei centroids and background, is calculated as the unknown area. Then, nuclei centroids are assigned differently as labels for each object. Markers are created by combining the unknown area and nuclei labeling image. In the markers, regions with known certainty (centroid and background) are labeled with distinct positive integers, while areas of uncertainty (unknown area) are left as zero. The labeled marker is treated as the lowest point in the image, and the watershed algorithm simulates water rising from this point gradually. At this time, boundaries are formed at overflow points, separating areas designated by different markers.

Let $M=\{m_{1},m_{2},\ldots ,m_{k}\}$ be a marker, where each $m_{i}$ is a label of pixels belonging to a specific marker area. *p* is the position of the unknown area excluding the background by the nuclei boundary image. The flooding simulation of the watershed starts with *p* around the marker. Water originating from each marker spreads to neighboring areas. The process of labeling the area at the *p* location starting from the *m_i_* marker is as follows:

(4) $$W(p)=\{\begin{array}{c} m_{i},(\forall n\in N_{\mathrm{labeled}}(p),W(n)=m_{i})\\ WL,(\exists n\in N_{\mathrm{labeled}}(p),W(n)\neq m_{i}) \end{array}$$

where W(p) represents the label at pixel *p* and $N_{\mathrm{labeled}}(p)$ represents the neighboring pixels that have already been labeled around *p*. *WL* is the watershed line that separates the two areas. The watershed algorithm labels each pixel by comparing it to the labels of neighboring pixels. The boundary of the area is formed at the point where the water meets.

In order to calculate the myotube diameter, it is important to understand where the myotube branches extend. However, it is difficult to identify the branch criteria of overlapping and growing myotubes using only myotube semantic segmentation generated through deep learning. We perform skeletonization on the myotube semantic segmentation to create a standard for identifying myotube branches (Fig. 2C).

Skeletonization is a process used to reduce a volumetric object to its simplest form, retaining only its essential structural features. In our approach, we implement a skeletonization method based on medial surface/axis thinning algorithms, which effectively transforms a complex shape into a skeletal representation. The process involves iteratively peeling off the outer layers of the object, aiming to preserve the medial axis, which represents the set of all points having more than one closest point on the object's boundary. The algorithm operates through a series of iterative sweeps across the image, methodically removing pixels until no further changes are observed in the image's structure.

### 2.5 Evaluation metrics

We calculated two evaluation metrics to evaluate the semantic segmentation results generated by deep learning. First, intersection over union (IoU), also known as the Jaccard Index, is a common metric used to evaluate the accuracy of object detection models, particularly in the context of segmentation tasks. IoU is calculated by dividing the area of overlap between the predicted segmentation (A) and the ground truth (B) by the area of union of the predicted and ground truth segmentations. Mathematically, it is expressed as

(5) $$\mathrm{IoU}=\frac{\left|A\cap B\right|}{\left|A\cup B\right|}=\frac{TP}{TP+FP+FN}$$

*TP* is true positive, the number of pixels correctly predicted as part of the object, and *FP* is false positive, the number of pixels of incorrectly predicted as part of the object. *FN* is false negative, the number of pixels that are part of the object but were not predicted as such. A higher IoU indicates a greater overlap and higher accuracy of the model's predictions.

The Dice coefficient is another crucial metric used for the evaluation of segmentation models. It is calculated by taking twice the area of overlap between the predicted and ground truth segmentations, divided by the sum of the areas of the predicted and ground truth segmentations. It can be formulated as

(6) $$\mathrm{Dice}=\frac{2|A\cap B|}{\left|A|+|B\right|}=\frac{2TP}{2TP+FP+FN}$$

The Dice coefficient is particularly useful in the medical imaging field, as it effectively captures the similarity in spatial dimensions between the predicted segmentation and the ground truth, making it ideal for evaluating models that predict the shapes of biological structures. A Dice coefficient of 1 indicates perfect and complete overlap, while a score of 0 indicates no overlap. In addition, we evaluated the model using 5-fold cross-validation.

## 3 Results

### 3.1 Myotube segmentation

**Figure 3.**
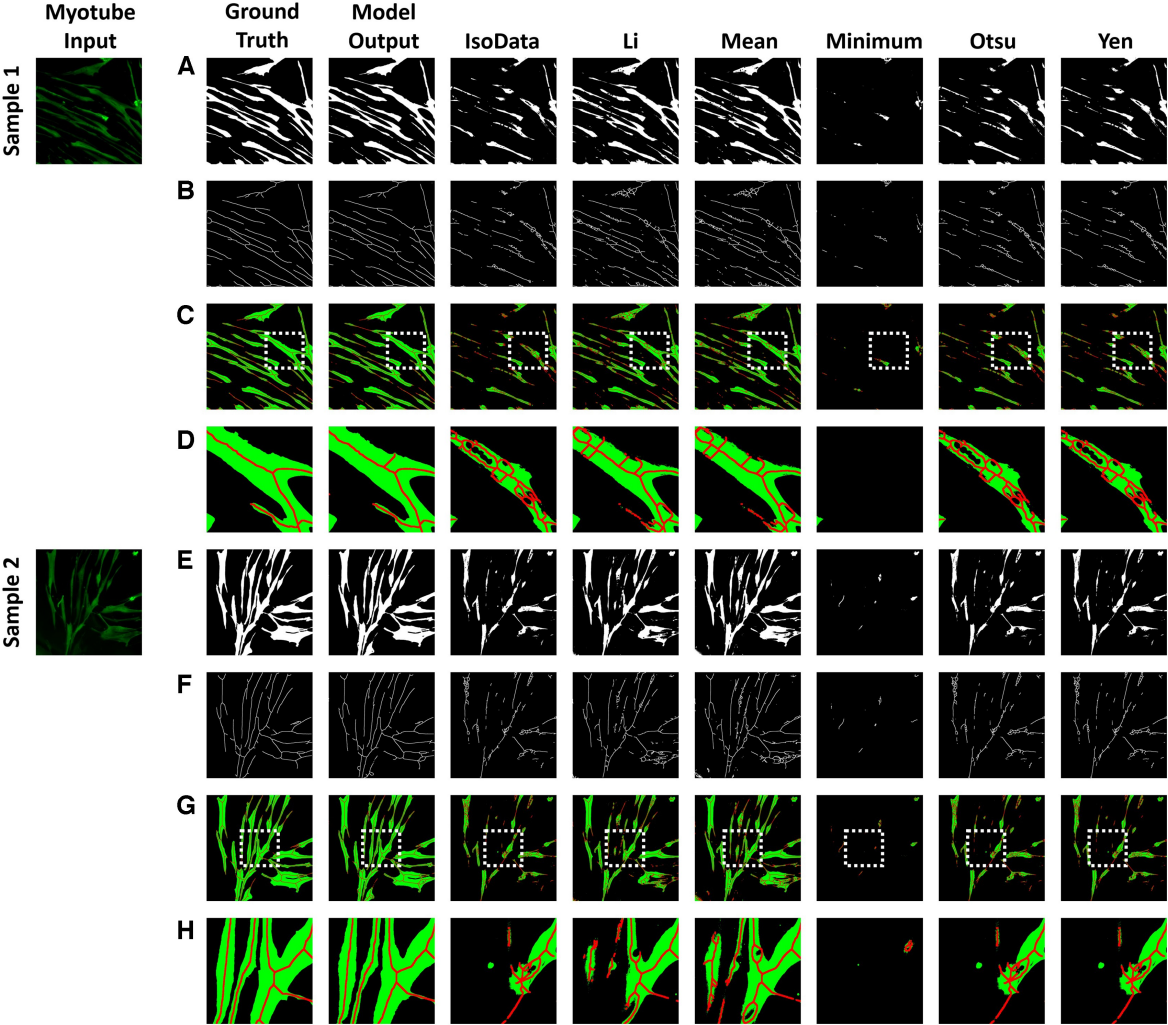
Myotube segmentation comparison of the proposed method with six threshold algorithms: IsoData, Li, Mean, Minimum, Otsu, and Yen. (A) and (E) are the results of myotube semantic segmentation, and (B) and (F) are the results of skeletonization from (A) and (E). (C) and (G) are the result of combining (A) and (B), (E) and (F). (D) and (H) are the results of enlarging the white box from (C) and (G).

We compared the results of various threshold algorithms to validate whether our proposed method accurately segmented the myotubes (Fig. 3). We selected six commonly used threshold algorithms: IsoData, Li, Mean, Minimum, Otsu, and Yen. In the case of the deep learning model results, it demonstrated semantic segmentation very similar to the ground truth. However, the threshold analysis results are greatly influenced by the quality of the input data, causing brighter parts of the image to eliminate darker areas, leaving regions not recognized as myotubes. Additionally, noise leads to images with uneven boundaries. Table 2 shows the results of calculating the IoU and Dice coefficient. The semantic segmentation generated from the deep learning model shows that both IoU and Dice coefficients are close to 1, showing that it is very similar to the ground truth. Threshold algorithms show overall low IoU and Dice coefficients. Applying skeletonization to myotube segmentation images results in more pronounced differences. Skeletonization is significantly affected by the continuity of boundaries and holes within the object. The results from the threshold algorithms display various branches caused by holes from nuclei or irregular boundaries. These branches can interfere with the calculation of the myotube diameter. In contrast, the results of the deep learning model show branch structures that are stretched in lines similar to the ground truth. This makes it a useful tool for identifying myotube branches and accurately calculating myotube diameters.

**Table 2.** Architecture of the deep-learning model for myotube segmentation.

Model	Deep learning	IsoData	Li	Mean	Minimum	Otsu	Yen
IoU	0.922	0.2939	0.6524	0.6559	0.1927	0.3905	0.3153
Dice coefficient	0.9553	0.4184	0.7741	0.7761	0.2787	0.5344	0.4232

### 3.2 Nuclei segmentation

We constructed and trained a model that could obtain the nuclei boundaries and centroids simultaneously; using these generated boundaries and centroids in the marker-controlled watershed algorithm, an image with well-separated overlapping boundaries can be obtained to accurately measure the number of nuclei. Table 3 shows the IoU and Dice coefficient for nuclei semantic segmentation and nuclei centroid images generated by the deep learning model. In both results, the IoU and Dice coefficients are close to 1, proving that the results are very similar to the ground truth.

**Table 3.** IoU and Dice coefficient of the nuclei semantic segmentation and nuclei centroid by deep learning model from 40 test images.

Model	Nuclei semantic segmentation	Nuclei centorid
IoU	0.9633	0.8786
Dice coefficient	0.9852	0.9394

**Figure 4.**
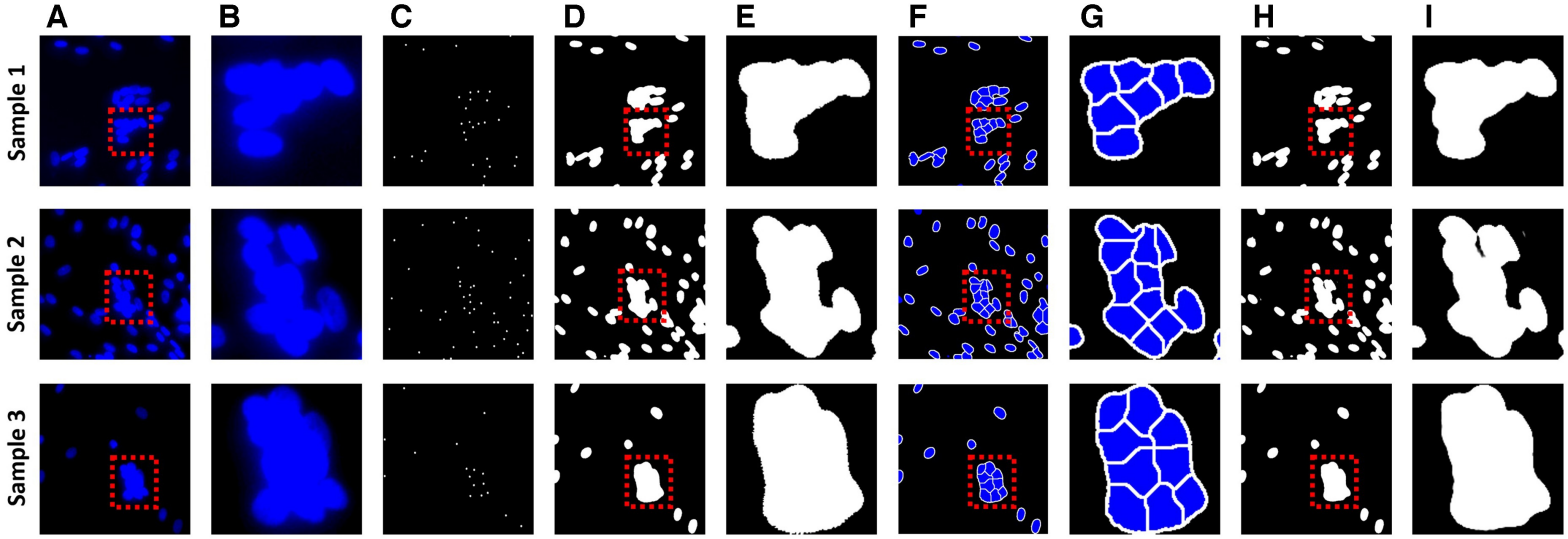
Nuclear segmentation results for counting the number of nuclei. (A) is the original nuclei image. (B) is an enlarged image of the box in (A). (C) and (D) are nuclei centroid images and nuclei semantic segmentation, which are the results of the deep learning model. (E) is an enlarged image of the box in (D). (F) is an image obtained by applying the marker-controlled watershed algorithm from (C) and (D). (G) is an enlarged image of the box in (F). (H) is a result trained to segment overlapped nuclei directly from deep learning without watershed. (I) is an enlarged image of the box in (H).

Figure 4 shows the results of the deep learning model, where the segmentation output is obtained by applying the marker-controlled watershed algorithm to the images with nuclei semantic segmentation and nuclei centroid. The model shows that it effectively distinguishes regions that are otherwise difficult to segment owing to overlapped nuclei. The results from training a deep learning model with nuclei segmentation labels, without using the watershed algorithm, show that segmentation of overlapped nuclei is impossible. During image segmentation, the deep learning model uses pooling layers to reduce the image's spatial dimension, decreasing computational demands and achieving spatial invariance. However, this also lowers the feature map resolution, impairing the model's ability to distinguish object boundaries. In contrast, the results using the watershed show the segmentation of regions of each nucleus in overlapped nuclei. The training goal of deep learning in the proposed model is to find binary and centroid, not accurate nuclei segmentation, and the outputs of deep learning are used as images to apply to the watershed algorithm. Therefore, consistent nuclei segmentation is possible and overlapped nuclei can also be effectively segmented. Combining deep learning and watershed makes it possible to accurately measure the number of nuclei, an indicator that determines the growth and state of myotubes.

### 3.3 Model comparison

**Figure 5.**
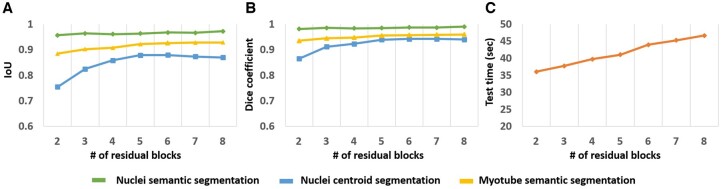
Segmentation evaluation results according to the number of residual blocks. The number of residual blocks is from 2 to 8. (A) is the result of IoU, (B) is the result of dice coefficient, and (C) is the test time required to generate 40 images.

Figure 5 shows the performance difference according to the number of residual blocks in the proposed model structure. We dealt with the optimal number of residual blocks by setting the number of residual blocks from 2 to 8 in the proposed model structure. We evaluated the model based on performance indicators (IoU, Dice coefficient) and efficiency (related to test time). As the number of residual blocks increased, more parameters were introduced to be calculated, so the test time increased continuously with the number of residual blocks. However, the performance indicators improved steadily only up to 5 residual blocks, and thereafter, the improvement was minimal. This indicates that increasing the number of residual blocks beyond 5 decreases the performance benefit and increases the computational burden unnecessarily.

**Figure 6.**
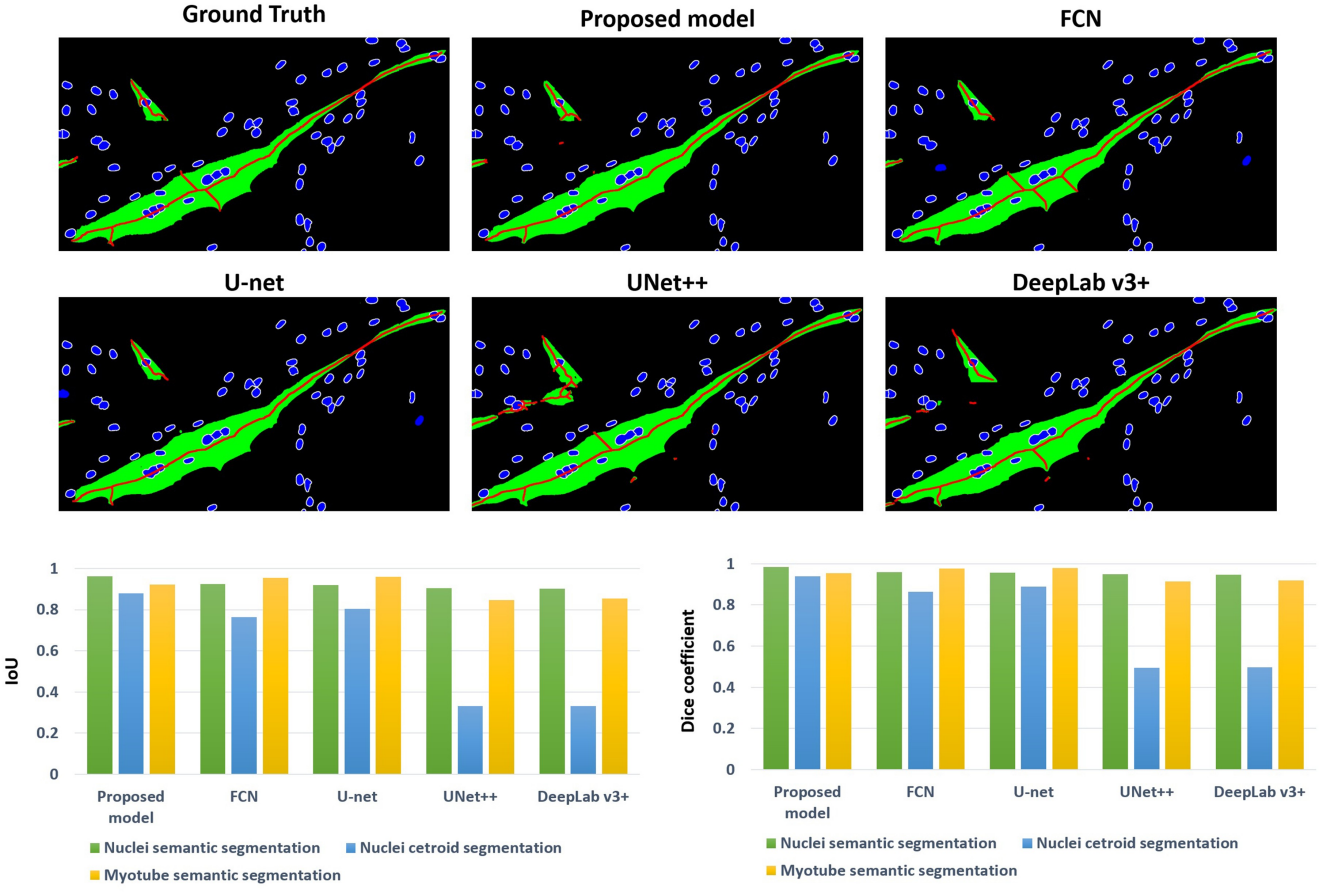
Comparison results with famous image segmentation models: FCN, U-net, UNet++, and DeepLab v3+. The result above is an image cropped from an image that underwent post-processing on the results of a deep learning model. The box graph below shows the segmentation performance results for each model.

Figure 6 shows the comparison results between our model and other well-known segmentation models, including FCN (Long *et al.* 2015) [35], U-net (Ronneberger *et al.* 2015) [27], U-net++ (Zhou *et al.* 2018) [36], and Deeplab v3+ (Chen *et al.* 2018) [37]. The models were trained to generate three outputs, the same as the proposed model. Looking at the results of the compared models, the nuclei semantic segmentation and myotube semantic segmentation results show similar results to the proposed model, but there is a big difference in the nuclei centroid images. The nuclei centroid images are the main criteria for distinguishing overlapped cells to measure the number of cell nuclei. If the centroid images are not generated properly, it is difficult to distinguish them even if the watershed algorithm is applied. On the other hand, the proposed model has three class layers at the end, and since the output is generated through a different class layer for each output, it can be confirmed that the centroids are generated well.

### 3.4 Dexamethasone-induced muscular atrophy in young and aged skMCs

First, we conducted FACS analysis to measure the levels of lipofuscin and ROS, and performed SA-β-Gal staining to verify the degree of aging in young and aged skeletal muscle cells (see Methods S2, S3 and Fig. S1 in Supplementary Information). SA-β-Gal is a widely used biomarker for cellular senescence, indicating aging cells. Reactive oxygen species (ROS) are chemically reactive molecules that increase with cellular stress and aging, leading to oxidative damage. Lipofuscin is an age-related pigment that accumulates in cells as a result of oxidative stress and lysosomal degradation. As a result, we found that the number of SA-β-Gal positive cells was 2.94-fold higher in aged muscle. Additionally, FACS analysis revealed higher levels of both lipofuscin and ROS in aged cells compared to young cells. Through these results, we confirmed that the aged cells exhibit characteristics of aging.

**Figure 7.**
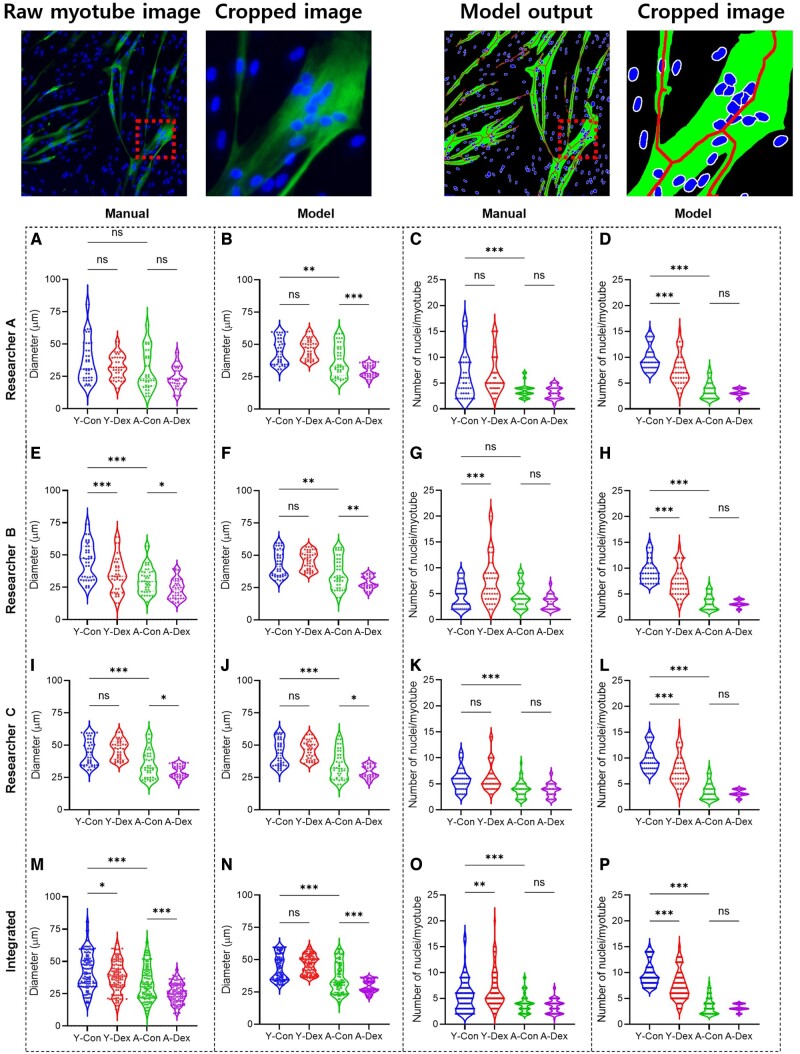
Original myotube image and model output image with cropped image. Manual and deep-learning-based morphological analyses as well as the effects of dexamethasone on young and aged skMCs. (A to P) Quantification of myotube features based on the diameter as manually assessed by researchers A−C (A, E, and I) and by model (B, F, and J) as well as nuclei as manually assessed by researchers A−C (C, G, and K) and by model (D, H, and L). (M to P) Integrated quantification results of the myotube features, both for diameter assessed manually (M) and by model (N) as well as for nuclei assessed manually (O) and by model (P). (*n* = 3) (*P < 0.05; **P < 0.01; ***P < 0.001; compared to young control, Y-Con; young control, Y-Dex; young with dexamethasone, A-Con; aged control, A-Dex; aged with dexamethasone).

We validated the accuracy of the proposed deep-learning model by comparing four distinct groups of cells: human primary young and aged skMCs, as well as the corresponding dexamethasone-treated groups. Each group used 10 fluorescence images. Using the developed algorithm, we analyzed morphological features such as the diameter of the myotube and the number of nuclei in the myotubes that represent myotube formation. Specifically, we compared the manual measurements of myotube features by three researchers with the results from the deep-learning model (Fig. 7).

Specifically, when comparing the results of all researchers, there were significant differences in the manual analysis between each researcher’s results, but with the help of the model, it was confirmed that all three researchers produced consistent results (Fig. 7). Furthermore, for the number of nuclei, the manual analysis showed considerable variation, but with the help of the model, we observed that this variation was significantly reduced. Additionally, when comparing the combined results of all researchers with the individual data from each researcher, there were significant differences in the manual analysis (Fig. 7M−P). However, with the assistance of the model, it was confirmed that similar results could be obtained. Notably, when comparing the manual diameter measurements by researchers A, B, and C (Table 4), there was a large difference between the two groups; however, similar results were obtained when using model assistance. This difference was also noted for nuclei counting (Table 5). In the manual counting, there was a significant discrepancy between the two measures, but consistent results were obtained with the deep-learning model. Ultimately, the data variance when combining the measurements made manually by both researchers and those obtained with model assistance was reduced significantly by the use of the deep-learning model.

**Table 4.** Comparison of myotube diameter measured by researchers A, B, and C in 10 samples for each group (Y-Con: young control, Y-Dex: young with dexamethasone, A-Con: aged control, A-Dex: aged with dexamethasone), where bold values represent the overall mean and variance for researchers A, B, and C.

Myotube diameter (μm)		w/model				w/o model			
		Y-Con	Y-Dex	A-Con	A-Dex	Y-Con	Y-Dex	A-Con	A-Dex
A	Avg	43.54	46.14	36.24	29.03	37.93	32.07	30.31	23.76
	Std	9.62	7.09	11.27	4.66	17.11	9.38	15.79	8.66
B	Avg	42.58	45.42	34.81	28.44	45.97	35.04	29.24	23.29
	Std	9.40	6.69	11.09	4.65	14.40	13.82	8.32	7.56
C	Avg	43.17	45.86	35.73	28.78	41.84	36.48	34.60	25.28
	Std	9.93	7.26	11.80	4.87	17.35	11.83	9.94	9.34
All	Avg	**43.10**	**45.81**	**35.59**	**28.75**	**42.16**	**34.59**	**31.72**	**24.15**
	Std	**9.66**	**7.02**	**11.41**	**4.73**	**16.44**	**11.77**	**12.28**	**8.52**

**Table 5.** Comparison of the number of nuclei measured by researchers A, B, and C in 10 samples for each group (Y-Con: young control, Y-Dex: young with dexamethasone, A-Con: aged control, A-Dex: aged with dexamethasone), where bold values represent the overall mean and variance for researchers A, B, and C.

The number of nuclei		w/model				w/o model			
		Y-Con	Y-Dex	A-Con	A-Dex	Y-Con	Y-Dex	A-Con	A-Dex
A	Avg	9.79	7.53	3.63	3.21	6.53	6.16	3.68	3.05
	Std	2.46	3.07	1.78	0.69	4.56	3.26	1.49	1.19
B	Avg	9.53	7.32	3.47	3.16	4.68	7.05	4.47	3.37
	Std	2.37	2.87	1.60	0.67	2.41	4.25	2.26	1.42
C	Avg	9.68	7.47	3.58	3.21	5.05	4.95	3.84	3.58
	Std	2.54	3.10	1.82	0.69	2.93	1.90	1.93	1.31
All	Avg	**9.67**	**7.44**	**3.56**	**3.19**	**5.44**	**6.32**	**4.00**	**3.36**
	Std	**2.46**	**3.01**	**1.74**	**0.69**	**3.50**	**3.64**	**1.91**	**1.31**

The impact of dexamethasone on general and aging muscles was more pronounced in the aged cells. According to the myotube diameter and length measurements, no significant difference was observed between the Young Con. vs. Young Dex. groups. However, in the Aged Con. vs. Aged Dex. group, a significant decrease was observed for the Dex group. Additionally, the number of nuclei per myotube also decreased more significantly in the aged skMCs than young skMCs, indicating that the atrophic effects of the drug were greater on the aged skMCs. Additionally, we verified the applicability of the model using an additional data set (atrophy and hypertrophy induced by electroceuticals) (see SI Results S1 and Fig. S2 in the Supplementary Information).

### 3.5 Expression of skeletal-muscle-related mRNA by qPCR

**Figure 8.**
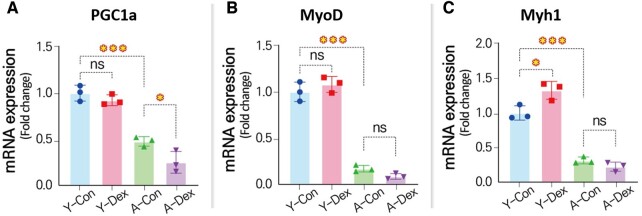
Changes in gene expressions related to skeletal muscle differentiation. mRNA expression levels for (A) PGC1a, (B) MyoD, and (C) Myh1 (*n* = 3) (*P < 0.05; **P < 0.01; ***P < 0.001; compared to young control).

We analyzed the expression of skeletal-muscle-related mRNA to validate the data generated by the deep-learning-model-based image processing approach in comparison with data obtained through conventional manual methods. Specifically, we examined the expression of PGC1a, a master regulator of mitochondrial biogenesis, oxidative metabolism, and muscle fiber type determination (Fig. 8A). PGC1a plays a critical role in maintaining the oxidative capacity of skeletal muscle, and protecting muscle cells from atrophy (O'Hagan *et al.* 2009). Dexamethasone, a glucocorticoid known to induce muscle atrophy, significantly downregulated PGC1a expression (Annie *et al.* 2019), particularly in aged skeletal muscle cells, where a 47.48% reduction was observed. This reduction in PGC1a impairs mitochondrial function, leading to decreased oxidative metabolism and energy production. The downregulation of PGC1a diminishes the muscle's ability to resist catabolic pathways, including those that degrade muscle proteins (Kang and Ji 2013). Furthermore, the diminished expression of PGC1a correlates with an increase in markers of muscle degradation, such as MuRF1 and Atrogin-1, highlighting the interplay between glucocorticoid signaling and mitochondrial health in the context of aging.

The parallel reduction in PGC1a expression, observed both in aged cells treated with dexamethasone and in our image-based analyses, underscores the consistency between the results obtained through the deep-learning approach and traditional manual methods. These findings highlight PGC1a’s central role in mediating the adverse effects of dexamethasone on skeletal muscle, particularly in aged cells, where mitochondrial dysfunction and muscle atrophy are more pronounced.

Similarly, the MyoD gene (Fig. 8B), which induces myotube differentiation, showed no difference in young skMCs but exhibited a 57.14% reduced expression in aged skMCs, suggesting that the atrophic effects of dexamethasone were maximized in the aged skMCs. Interestingly, the Myh1 gene showed an opposite trend, with increased expression in the Dex group in young skMCs and a less pronounced decrease in the aged group. In conclusion, our study underscores the heightened vulnerability of aged skMCs to the adverse effects of dexamethasone compared to younger cells. Aged skMCs exhibited a more pronounced response to the drug's side effects, evident in parameters such as myotube diameter, nuclei count, and gene expression levels. Our findings, corroborated by both qPCR analysis and our innovative deep-learning-based image processing method, highlight the need for tailored therapeutic approaches to mitigate the detrimental effects of dexamethasone, particularly in the elderly.

## 4 Conclusion

The present study successfully developed a deep-learning-based method for the simultaneous segmentation of myotubes and nuclei from fluorescence images. Our deep learning model, which outputs myotube semantic segmentation, nuclei semantic segmentation, and nuclei centroid, has proven effective in accurately distinguishing overlapped nuclei by applying the watershed algorithm, and identifying the branches of the myotube through skeletonization to calculate the myotube diameter. The measurements of myotube diameter, length, and number of nuclei derived from these outputs demonstrated superior consistency and reliability compared to traditional methods conducted by human experimenters. The implementation of this deep learning approach significantly accelerates the analysis process, enhances measurement accuracy, and reduces resource expenditure. These improvements are particularly beneficial for advancing drug development targeting muscle-related diseases such as sarcopenia, which currently lack effective treatments. The enhanced precision and efficiency of our proposed system are expected to make significant contributions to muscle research and pharmacological screening, offering a robust tool for the development of new therapeutic strategies. The promising results of this study suggest that deep learning-based image segmentation can play a crucial role in streamlining drug development processes and improving our understanding of muscle biology. Future work may expand on these findings to explore additional applications and further refine the methodology for broader use in biomedical research.

## Author contributions

S.P. and M.K. conceptualized the study and performed investigation. J.J. and S.Y. curated the data. S.P. designed the methodology and wrote the codes and software. S.P. and M.K. work in validation and visualization as well as writing—original draft. M.S.K. and I.M. are the roles in funding acquisition, project administration, supervision, and manuscript writing—review & editing

## Supplementary Material

btae658_Supplementary_Data

## Data Availability

The data underlying this article are available at https://github.com/tdn02007/QA-skMCs-Seg.

## References

[btae658-B1] Al-Kofahi Y , Zaltsman A, Graves R et al A deep learning-based algorithm for 2-D cell segmentation in microscopy images. BMC Bioinformat 2018;19:365. 10.1186/s12859-018-2375-z PMC6171227 30285608

[btae658-B2] Annie L , Gurusubramanian G, Roy VK. Dexamethasone mediated downregulation of PGC-1α and visfatin regulates testosterone synthesis and antioxidant system in mouse testis. Acta Histochem 2019;121:182–8. 30579591 10.1016/j.acthis.2018.12.004

[btae658-B3] Beucher S , Meyer F. The morphological approach to segmentation: the watershed transformation. In: Dougherty ER (ed.). Mathematical Morphology in Image Processing. New York: CRC Press, 1992, 433–81.

[btae658-B4] Brown JC , Harhay MO, Harhay MN. Sarcopenia and mortality among a population-based sample of community-dwelling older adults. J Cachexia Sarcopenia Muscle 2016;7:290–8. 27239410 10.1002/jcsm.12073 PMC4864252

[btae658-B5] Carpenter AE , Jones TR, Lamprecht MR et al CellProfiler: image analysis software for identifying and quantifying cell phenotypes. Genome Biol 2006;7:R100–11. 17076895 10.1186/gb-2006-7-10-r100 PMC1794559

[btae658-B6] Chandrasekaran SN , Ceulemans H, Boyd JD et al Image-based profiling for drug discovery: due for a machine-learning upgrade? Nat Rev Drug Discov 2021;20:145–59. 33353986 10.1038/s41573-020-00117-w PMC7754181

[btae658-B7] Chen LC , Zhu Y, Papandreou G et al Encoder-decoder with atrous separable convolution for semantic image segmentation. In: Ferrari V, Hebert M, Sminchisescu C et al. (eds.), *Proceedings of the European Conference on Computer Vision (ECCV)*. Munich, Germany, Cham: Springer, 2018, 801–18.

[btae658-B8] Frontera WR , Ochala J. Skeletal muscle: a brief review of structure and function. Calcif Tissue Int 2015;96:183–95. 25294644 10.1007/s00223-014-9915-y

[btae658-B9] Ghaznavi A , Rychtáriková R, Saberioon M et al Cell segmentation from telecentric bright-field transmitted light microscopy images using a residual attention U-Net: a case study on HeLa line. Comput Biol Med 2022;147:105805. 35809410 10.1016/j.compbiomed.2022.105805

[btae658-B10] Gu S , Wen C, Xiao Z et al MyoV: a deep learning-based tool for the automated quantification of muscle fibers. Brief Bioinform 2024;25:bbad528. 10.1093/bib/bbad528 PMC10810329 38271484

[btae658-B11] He K , Zhang X, Ren S et al Deep residual learning for image recognition. In: *Proceedings of the IEEE Conference on Computer Vision and Pattern Recognition*. Las Vegas, NV, USA. Washington, D.C., USA: IEEE, 2016, 770–8.

[btae658-B12] Hollandi R , Szkalisity A, Toth T et al nucleAIzer: a parameter-free deep learning framework for nucleus segmentation using image style transfer. Cell Syst 2020;10:453–8.e6. 34222682 10.1016/j.cels.2020.04.003 PMC8247631

[btae658-B13] Jeong GJ , Castels H, Kang I et al Nanomaterial for skeletal muscle regeneration. Tissue Eng Regen Med 2022;19:253–61. 35334091 10.1007/s13770-022-00446-4 PMC8971233

[btae658-B14] Jesinkey SR , Korrapati MC, Rasbach KA et al Atomoxetine prevents dexamethasone-induced skeletal muscle atrophy in mice. J Pharmacol Exp Ther 2014;351:663–73. 25292181 10.1124/jpet.114.217380 PMC4244586

[btae658-B15] Kang C , Ji LL. Role of PGC-1α in muscle function and aging. J Sport Health Sci 2013;2:81–6.

[btae658-B16] Kim E , Park S, Hwang S et al Deep learning-based phenotypic assessment of red cell storage lesions for safe transfusions. IEEE J Biomed Health Inform 2022;26:1318–28. 34388103 10.1109/JBHI.2021.3104650

[btae658-B17] Kim H , Cho SC, Jeong H-J et al Indoprofen prevents muscle wasting in aged mice through activation of PDK1/AKT pathway. J Cachexia Sarcopenia Muscle 2020;11:1070–88. 32096917 10.1002/jcsm.12558 PMC7432593

[btae658-B18] Kim MY , Shin HY, Cho SC et al Silver electroceutical technology to treat sarcopenia. Proc Natl Acad Sci USA 2023;120:e2300036120. 37549292 10.1073/pnas.2300036120 PMC10438839

[btae658-B19] Krizhevsky A , Sutskever I, Hinton GE. ImageNet classification with deep convolutional neural networks. Commun ACM 2017;60:84–90.

[btae658-B20] Kwak JY , Kwon KS. Pharmacological interventions for treatment of sarcopenia: current status of drug development for sarcopenia. Ann Geriatr Med Res 2019;23:98–104. 32743297 10.4235/agmr.19.0028 PMC7370765

[btae658-B21] Lee TC , Kashyap RL, Chu CN. Building skeleton models via 3-D medial surface axis thinning algorithms. CVGIP 1994;56:462–78.

[btae658-B22] Li Y , Yang Z, Wang Y et al A neural network approach to analyze cross-sections of muscle fibers in pathological images. Comput Biol Med 2019;104:97–104. 30463027 10.1016/j.compbiomed.2018.11.007 PMC6318808

[btae658-B23] Long FX. Microscopy cell nuclei segmentation with enhanced U-Net. BMC Bioinformat 2020;21:8. 10.1186/s12859-019-3332-1 PMC6950983 31914944

[btae658-B24] Long J , Shelhamer E, Darrell T. Fully convolutional networks for semantic segmentation. In: *2015 IEEE Conference on Computer Vision and Pattern Recognition (CVPR), Boston, MA, USA*. Washington, D.C., USA: IEEE, 2015; 3431–40. 10.1109/TPAMI.2016.2572683 27244717

[btae658-B25] Morley JE. Treatment of sarcopenia: the road to the future. J Cachexia Sarcopenia Muscle 2018;9:1196–9. 30697982 10.1002/jcsm.12386 PMC6351669

[btae658-B26] Nair KS. Aging muscle. Am J Clin Nutr 2005;81:953–63. 15883415 10.1093/ajcn/81.5.953

[btae658-B27] Noe S , Corvelyn M, Willems S et al The myotube analyzer: how to assess myogenic features in muscle stem cells. Skelet Muscle 2022;12:12. 35689270 10.1186/s13395-022-00297-6 PMC9185954

[btae658-B28] O'Hagan KA , Cocchiglia S, Zhdanov AV et al PGC-1α is coupled to HIF-1α-dependent gene expression by increasing mitochondrial oxygen consumption in skeletal muscle cells. Proc Natl Acad Sci USA 2009;106:2188–93. 19179292 10.1073/pnas.0808801106 PMC2632715

[btae658-B29] Otsuka Y , Egawa K, Kanzaki N et al Quercetin glycosides prevent dexamethasone-induced muscle atrophy in mice. Biochem Biophys Rep 2019;18:100618. 30805562 10.1016/j.bbrep.2019.100618 PMC6372881

[btae658-B30] Poletti E , Zappelli F, Ruggeri A et al A review of thresholding strategies applied to human chromosome segmentation. Comput Methods Programs Biomed 2012;108:679–88. 22261220 10.1016/j.cmpb.2011.12.003

[btae658-B31] Qin J , Du R, Yang YQ et al Dexamethasone-induced skeletal muscle atrophy was associated with upregulation of myostatin promoter activity. Res Vet Sci 2013;94:84–9. 22939086 10.1016/j.rvsc.2012.07.018

[btae658-B32] Ronneberger O , Fischer P, Brox T. U-Net: convolutional networks for biomedical image segmentation. Lect Notes Comput Sci 2015;9351:234–41.

[btae658-B33] Sadanandan SK , Ranefall P, Le Guyader S et al Automated training of deep convolutional neural networks for cell segmentation. Sci Rep 2017;7:7860. 28798336 10.1038/s41598-017-07599-6 PMC5552800

[btae658-B34] Seene T , Kaasik P. Role of exercise therapy in prevention of decline in aging muscle function: glucocorticoid myopathy and unloading. J Aging Res 2012;2012:172492. 22778959 10.1155/2012/172492 PMC3385633

[btae658-B35] Sobestiansky S , Michaelsson K, Cederholm T. Sarcopenia prevalence and associations with mortality and hospitalisation by various sarcopenia definitions in 85-89 year old community-dwelling men: a report from the ULSAM study. BMC Geriatr 2019;19:318. 31747923 10.1186/s12877-019-1338-1 PMC6864927

[btae658-B36] Toptas M , Yalcin M, Akkoc I et al The relation between sarcopenia and mortality in patients at intensive care unit. Biomed Res Int 2018;2018:5263208. 29789798 10.1155/2018/5263208 PMC5896340

[btae658-B37] Walston JD. Sarcopenia in older adults. Curr Opin Rheumatol 2012;24:623–7. 22955023 10.1097/BOR.0b013e328358d59b PMC4066461

[btae658-B38] Wu J , Yuan T, Zeng J et al A medically assisted model for precise segmentation of osteosarcoma nuclei on pathological images. IEEE J Biomed Health Inform 2023;27:3982–93. 37216252 10.1109/JBHI.2023.3278303

[btae658-B39] Xiao C , Wang J, Yang S et al VISN: virus instance segmentation network for TEM images using deep attention transformer. Brief Bioinform 2023;24:bbad373. 37903415 10.1093/bib/bbad373

[btae658-B40] Zhou Z , Rahman Siddiquee MM, Tajbakhsh N et al Unet++: a nested u-net architecture for medical image segmentation. In: Stoyanov D, Taylor Z, Carneiro G et al. (eds.), Deep Learning in Medical Image Analysis and Multimodal Learning for Clinical Decision Support. Vol. 11045, Granada, Spain, Cham: Springer, 2018, 3–11. 32613207 10.1007/978-3-030-00889-5_1 PMC7329239

