# Supplementary material for: Quantitative analysis of the dexamethasone side effect on human-derived young and aged skeletal muscle by myotube and nuclei segmentation using deep learning
Source: Bioinformatics. 2025 Jan 3;41(1):btae658. doi: 10.1093/bioinformatics/btae658 (PMC11723526; doi:10.1093/bioinformatics/btae658)
Supplement: btae658_Supplementary_Data [file btae658_supplementary_data.zip › Supplementary-Information-BI-re-fv3.docx]

**Supplementary Information (SI)**

**SI Methods**

**1. Human primary young and aged skeletal muscle culture and biomolecular analysis**

Primary skMCs (male donors aged 17 and 68 years) were purchased from Cook Myosite (SK-1111, LOT-01052-17M, 01013-68M). The cells were grown in 24-culture plates in a myotonic basal medium (MB-2222, Cook Myosite) containing 10% myotonic growth supplement (MB-3333, Cook Myosite) and 1% antibiotic-antimycotic solution 100X (SV30079.01, HyCloneTM). After 2 days of proliferation, the basal medium was replaced with a myotonic differentiation medium (MB-5555, Cook Myosite) supplemented with antibiotics for 4 days. Thereafter, the differentiation medium was changed to another medium containing 100 µM of dexamethasone for 1 day. Finally, the medium was replaced with fresh differentiation medium for one more day before harvesting and staining the cells for evaluation.

The myotubes were fixed in 4% formaldehyde (28906, Thermo) in phosphate-buffered saline (PBS) for 15 min. Following fixation, the samples were washed in PBS and permeabilized with 0.5% Triton X-100 (Sigma). Primary antibodies (MF-20, DSHB) were then applied at 1:200 for 2 h at room temperature prior to applying fluorescently labeled secondary antibodies (A21202, Invitrogen) at 1:100 for 1 h. Finally, a mounting medium with DAPI (H-1200, Vector Laboratories, Inc.) was applied, and images of size 2048×2048 pixels were acquired using a Nikon Ti2 confocal microscope.

RNA was manually isolated from the myotubes using the QIAzol lysis reagent (79306, QIAZEN). cDNA was reverse transcribed using TOPscriptTM RT DryMIX (RT100, Enzynomics) on a Biometra T One 96G (Analytik Jena).

Quantitative polymerase chain reaction (qPCR) was performed on a StepOnePlus (Applied Biosystems) using the KAPA SYBR FAST qPCR Master Mix 2X (KK4602, Kapa Biosystems) according to manufacturer instructions. The 2-∆∆Ct method was used to determine gene expression changes using beta-actin as the endogenous control.

**2. Analysis of Lipofuscin and ROS Expression Using Fluorescence-Activated Cell Sorting (FACS)**

We utilized the FACSAria III system (BD) to examine the differential expression of lipofuscin and ROS levels in young and aged skeletal muscle cells. For this analysis, we prepared a cell suspension of human Aged skeletal muscle cells (hAskMC) and human Young skeletal muscle cells (hYskMC) in a FACS buffer (eBioscience™ Flow Cytometry Staining Buffer, Invitrogen). Lipofuscin, an autofluorescent material, exhibits an excitation range of 360-480 nm, peaking at approximately 430 nm. Thus, we used the FITC channel for its detection. To assess ROS levels, cells were stained with 5 µM CELLROX™ Green (Invitrogen) and incubated for 30 minutes in complete culture media at 37°C, with subsequent ROS detection also using the FITC channel. Prior to each cell sorting session, the FACSAria III system was calibrated following the manufacturer's guidelines. The prepared cell suspension was then loaded into the system, and fluorescence intensity was analyzed. Data analysis was performed using BD FACSDiva software.

**3. Senescence-Associated β-Galactosidase (SA-β-Gal) Assay**

SA-β-Gal staining were conducted on the HiTESS substrate 24 hours after ES application. SA-β-Gal staining was performed according to the manufacturer’s protocol using an SA-β-Gal kit (Cell Signaling Technology). SA-β-Gal expression increases proportionally with cell aging. Following staining, images were acquired using an inverted microscope (Leica) for quantification. The number of SA-β-Gal-positive myotubes was then analyzed using IMARIS software.


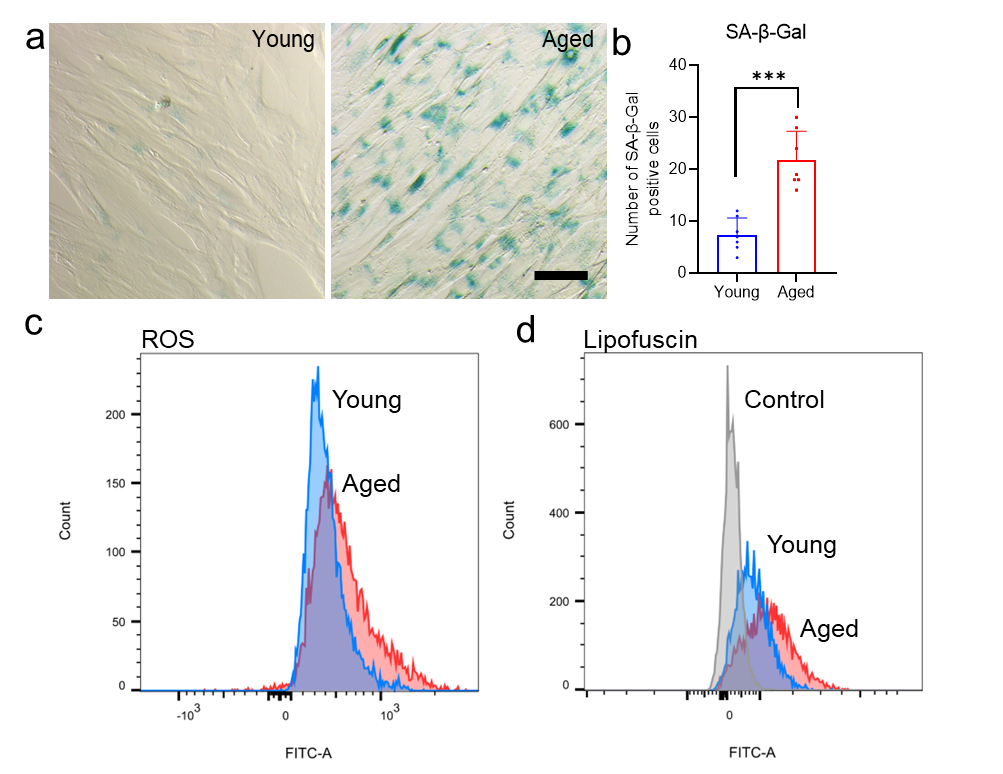


**Figure S1. Characterization of human-derived young and aged skeletal muscle cells.** (a, b) SA-Beta gal staining image of young and aged skMC (a) and quantification (b) (Scale bar 50 μm) (P-values denoted by * P<0.05; ** P<0.01; *** P<0.001). (c, d) Identification of ROS and lipofuscin using flow cytometry.

**SI Results**

**1. Performance Evaluation of the Model Using Additional Data Set**

We conducted a test using an additional image set to evaluate the performance of the model (see Figure S2). This image set involves applying electrical stimulation to young muscle cells to induce Hypertrophy (50 Hz) and Atrophy (500 Hz), followed by analyzing the diameter of the myotubes and the number of nuclei per myotube (Kim et al. 2023). The results showed that manual analysis could clearly distinguish between the Y-control and the 50 Hz Hypertrophy condition, but it was challenging to differentiate the 500 Hz Atrophy condition (Figure S2 A-C). However, with the help of the model, we were able to clearly distinguish the 500 Hz Atrophy condition (Figure S2 E-G). Additionally, there was minimal variation between researchers' analyses across all conditions, whether using manual or model-assisted methods (Figure S2 D and H). Regarding the number of nuclei, both manual and model analyses demonstrated statistically significant results, with no substantial differences between the two methods (Figure S2 I-P). In conclusion, through additional data analysis, we have demonstrated that the developed model is applicable not only to data generated from drug tests but also to data obtained from physical stimulation. We believe these results have the potential for broad applications in the development of drugs and therapies for treating muscular disorders in the future.


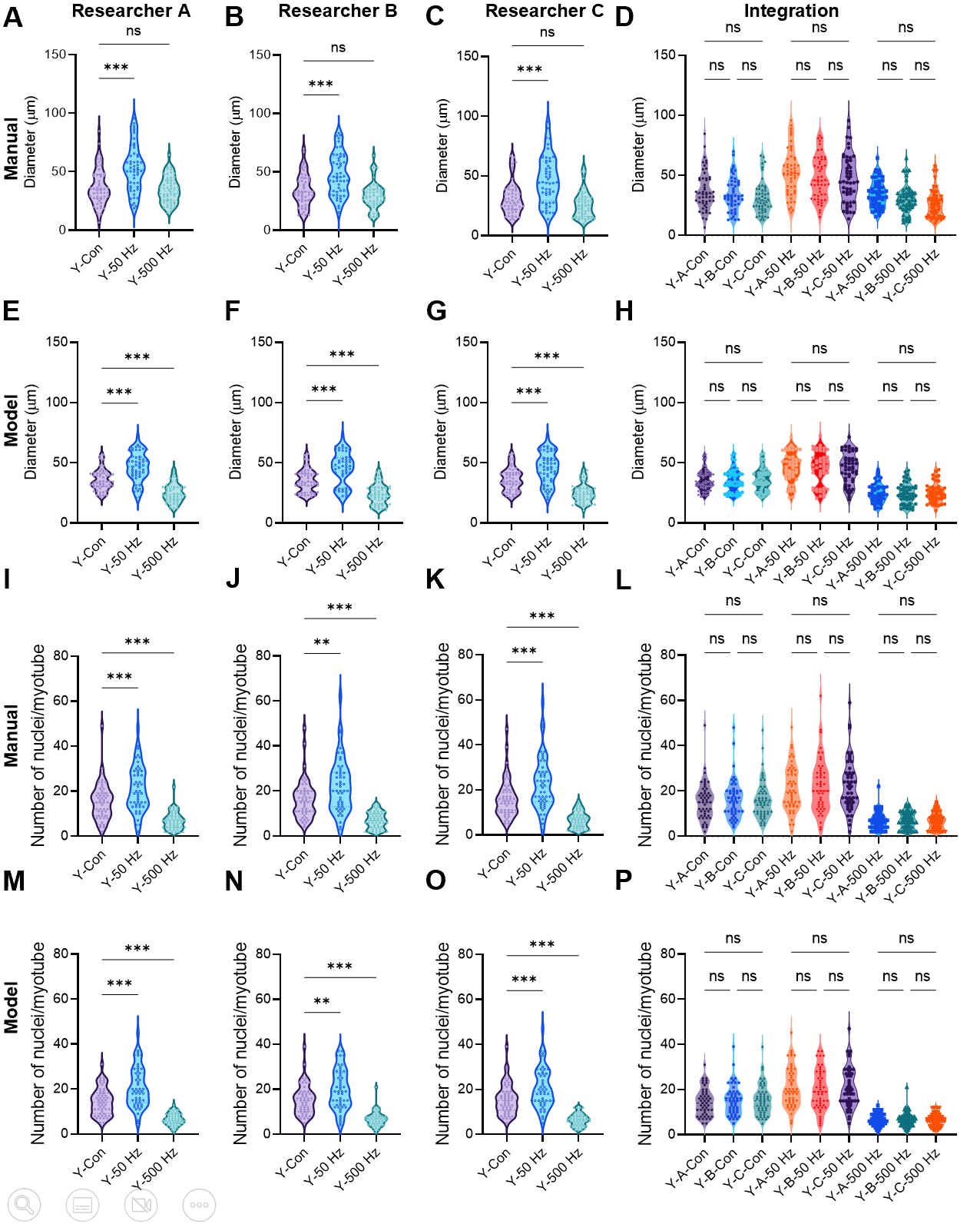


**Figure S2. Manual and deep-learning-based morphological analyses as well as the effects of electroceutical on young skMCs.** (A to H) Quantification of myotube features based on the diameter as manually assessed by researcher A-C (A to D) and by model (E to H). (I to P) Quantification of myotube features based on the number of nuclei/myotube as manually assessed by researcher A-C (I to L) and by model (M to P). (* P < 0.05; ** P < 0.01; *** P < 0.001;)

**Reference**

Kim MY, Shin HY, Cho SC et al. Silver electroceutical technology to treat sarcopenia. Proceedings of the National Academy of Sciences 2023;120:e2300036120.
