# Supplementary figures and images for: Quantitative analysis of the dexamethasone side effect on human-derived young and aged skeletal muscle by myotube and nuclei segmentation using deep learning
Source: Bioinformatics. 2025 Jan 3;41(1):btae658. doi: 10.1093/bioinformatics/btae658 (PMC11723526; doi:10.1093/bioinformatics/btae658)

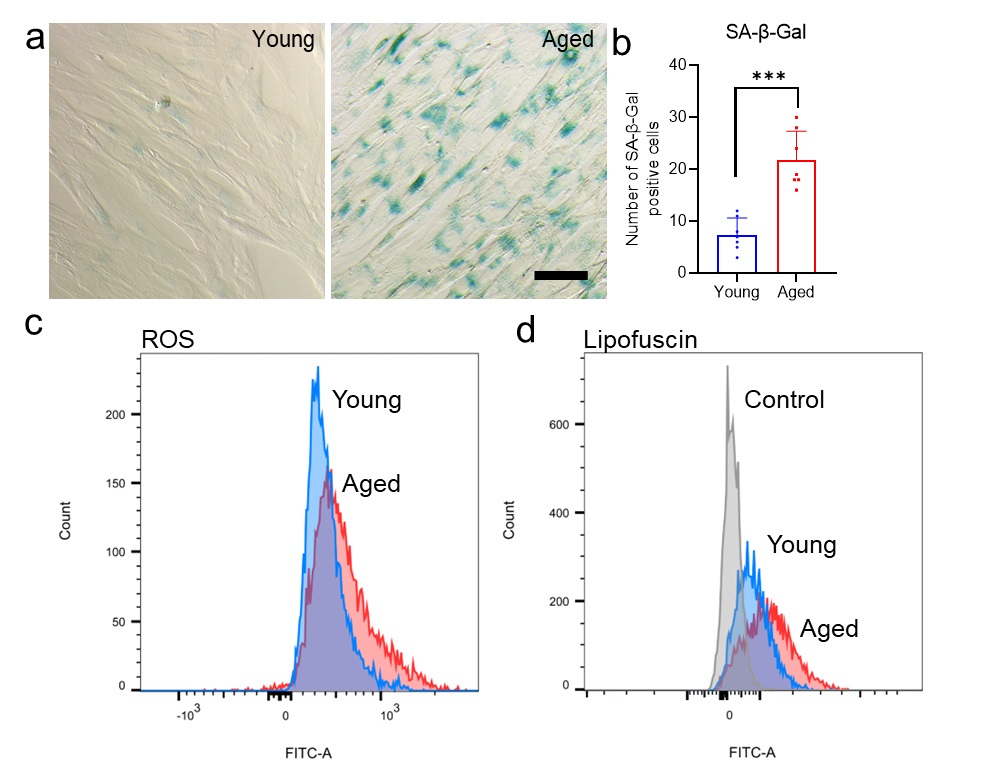

Supplement: btae658_Supplementary_Data [file btae658_supplementary_data.zip › Figure S1.jpg]

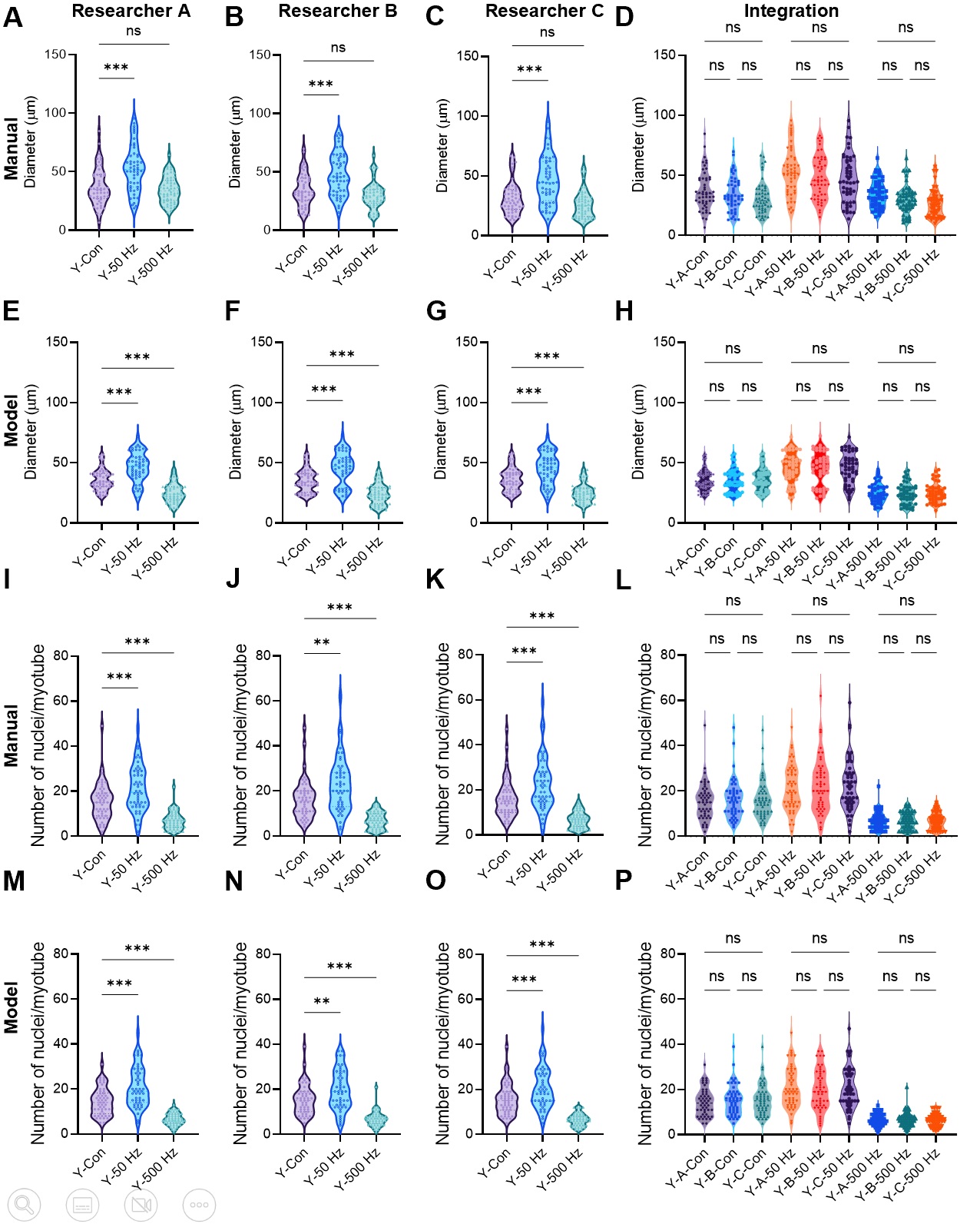

Supplement: btae658_Supplementary_Data [file btae658_supplementary_data.zip › Figure S2.jpg]
